# Revealing spatiotemporal transmission patterns and stages of COVID-19 in China using individual patients’ trajectory data

**DOI:** 10.1007/s43762-021-00009-8

**Published:** 2021-06-04

**Authors:** Tao Cheng, Tianhua Lu, Yunzhe Liu, Xiaowei Gao, Xianghui Zhang

**Affiliations:** SpaceTimeLab, Department of Civil, Environmental & Geomatic Engineering, University College London, Gower Street, London, WC1E 6BT UK

**Keywords:** Viral transmission, COVID-19, Patient trajectory, Spatiotemporal data mining

## Abstract

Gauging viral transmission through human mobility in order to contain the COVID-19 pandemic has been a hot topic in academic studies and evidence-based policy-making. Although it is widely accepted that there is a strong positive correlation between the transmission of the coronavirus and the mobility of the general public, there are limitations to existing studies on this topic. For example, using digital proxies of mobile devices/apps may only partially reflect the movement of individuals; using the mobility of the general public and not COVID-19 patients in particular, or only using places where patients were diagnosed to study the spread of the virus may not be accurate; existing studies have focused on either the regional or national spread of COVID-19, and not the spread at the city level; and there are no systematic approaches for understanding the stages of transmission to facilitate the policy-making to contain the spread.

To address these issues, we have developed a new methodological framework for COVID-19 transmission analysis based upon individual patients’ trajectory data. By using innovative space–time analytics, this framework reveals the spatiotemporal patterns of patients’ mobility and the transmission stages of COVID-19 from Wuhan to the rest of China at finer spatial and temporal scales. It can improve our understanding of the interaction of mobility and transmission, identifying the risk of spreading in small and medium-sized cities that have been neglected in existing studies. This demonstrates the effectiveness of the proposed framework and its policy implications to contain the COVID-19 pandemic.

## Introduction

Since the first case of COVID-19 was confirmed in December 2019 in Wuhan, China, over 134 million people have been infected with the disease and it has caused nearly 2.9 million deaths in 190 countries or regions, as of April 2021 (World Health Organisation, 2021). The pandemic has also triggered a variety of extreme restrictions, such as large-scale regional/national lockdowns and other non-pharmaceutical interventions (NPIs), with the global economy facing a recession (World Bank, 2020).

Researchers have devoted themselves extensively to analysing the characteristics of COVID-19 from multidisciplinary perspectives, including but not limited to its epidemiological and genomic characterisations (Lu et al., 2020), clinical features (Guan et al., 2020; Vetter et al., 2020), incubation period (Backer, Klinkenberg, & Wallinga, 2020), and asymptomatic carriers (Bai et al., 2020). Such studies have made valuable contributions to the treatments and vaccines used to suppress the disease (Kaur & Gupta, 2020; Kupferschmidt & Cohen, 2020). Moreover, substantial evidence collected during the outbreak in Wuhan showed that one of the typical modes of COVID-19 transmission is person to person interaction (Chan et al., 2020), underlining the fact that the large-scale and dispersed migration of the population can amplify a localised outbreak into a widespread pandemic (Balcan et al., 2009; Halloran et al., 2014). Therefore, in order to contain the spread of COVID-19, it is important to estimate human mobility and gauge its relationship with the viral transmission pattern. This task has aroused much attention not only in academia but also in governmental sectors pursuing evidence-based policy-making (Raboisson & Lhermie, 2020).

With the development of Internet of Things (IoT), mobile phones, and other Internet facilities, many studies have utilised mobile devices as a digital proxy for human mobility (Balzotti, Bragagnini, Briani, & Cristiani, 2018; Xie, Song, Li, & Ma, 2018). With regard to COVID-19, human mobility patterns derived from mobile devices/apps have been explored in relation to the severity of COVID-19 in an area, usually represented by the number of confirmed cases/deaths. It is vital to quantify changes in human mobility and understand the human mobility patterns during the pandemic at the national, regional, and individual levels (Grantz et al., 2020). At the national and regional levels, such work is helpful in assessing the effectiveness of governmental NPIs (e.g., lockdowns), helping policymakers to decide if extra or different interventions might be required (Cheng, Liu, Zhang, Dong, & Liu, 2021). At the individual level, understanding the human mobility pattern helps us to gain insight into people’s pattern of social contact and thus improve contact tracing (Yabe et al., 2020).

Nationwide mobile phone data were used to track the population outflow from Wuhan at the prefecture level, which has a high correlation with the cumulative number of infections (Jia et al., 2020). Badr et al. (2020) and Xiong, Hu, Yang, Luo, and Zhang (2020) employed daily mobile phone data to represent the population’s movement patterns for each county in the US, showing a positive correlation with the COVID-19 growth rate ratio. Moreover, introducing restrictions on the mobility of infected patients or on citizens in high-risk regions (such as lockdown) could delay and decrease the peak of the epidemic in the early stage of a COVID-19 outbreak, as examined in Shenzhen (Zhou et al., 2020), Tokyo (Yabe et al., 2020), and Italy and France (Santamaria et al., 2020). However, it was also found that restricting human mobility has only limited value, given that spatial heterogeneity has been evidenced in counties in the US (Hu et al., 2021) and in regions of the UK (Cheng et al., 2021).

Although a strong positive correlation exists between the mobility of the general public (represented by the digital proxy) and the transmission pattern of the coronavirus, there are limitations to the current studies. As warned of by Grantz et al. (2020), at the population level evidence has shown that using data derived from mobile phones may only partially reflect the movement of individuals, with the exact representativeness of such a measurement varying due to the different settings and uncertainties. Furthermore, only confirmed and asymptomatic patients and not the total floating population are the carriers and spreaders of the virus, but there has been no specific study on patients’ mobility. Moreover, confirmed case data only capture data when a particular person tests positive for coronavirus (i.e., becomes a ‘patient’) and the location where they are eventually tested (i.e., the destination of their journey before isolation/treatment). Hence, the virus may have already been transmitted to others in other locations, since the virus carrier could be still in the incubation period or asymptomatic. Therefore, not only the place where the patent was diagnosed but also the other places where they visited before the diagnosis should be investigated in order to gain a full picture of the virus transmission.

Last but not least, studies investigating the relation between human mobility/flow and COVID-19 transmission have mainly focused on their associations at the regional or national level. There is no systematic approach to identify if an outbreak is considered local or regional, if a city is at high risk due to having a high virus exposure, and what the next stages of the spread consist of. All these factors have strong policy implications. If outbreaks are local, then local lockdown or full testing might be more effective. If a city is at high risk, then it is likely that the number of patients will increase, so more preparations and resources are needed.

To overcome these limitations, this paper develops a new methodological framework for COVID-19 transmission analysis based upon individual patients’ trajectory data. Using innovative spatiotemporal data mining techniques, patients’ mobility patterns in cities during their incubation periods will be analysed. The spatiotemporal activity hotspots of patients will be detected, revealing the order and stages of the dynamic transmission of the virus. We believe that the deeper insights gained at the finer spatial and time scales will facilitate the creation of effective policies to contain the virus.

The paper is organised as follows. After the introduction in this section, Section 2 presents our data sources and pre-processing steps. Then, the methodology is presented in Section 3, including steps for analysing patients’ travel behaviours to revealing the diffusion stages and orders of the virus in space and time. The analytical results of the case are presented and discussed in detail in Section 4. Section 5 summarises the major findings and limitations of this study and makes suggestions for further research.

## Data and pre-processing

### Data introduction

**Fig. 1:**
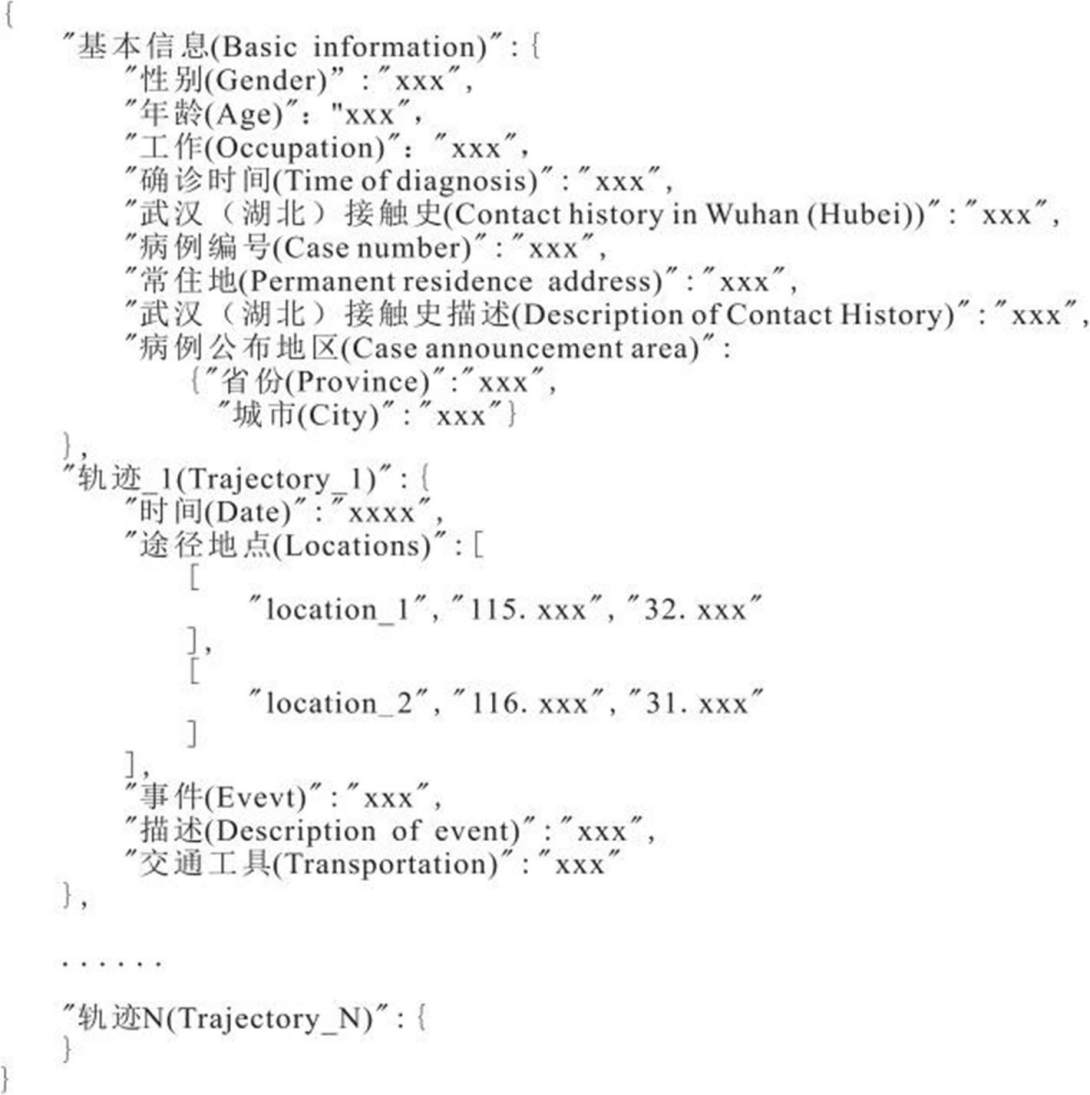
Example of raw data of BCBD

This study uses the trajectory data of patients confirmed to have COVID-19 in mainland China, primarily focusing on the early stage of this pandemic. Two data sources, patient trajectory data and confirmed case data from China last spring, are used in this study. The patients’ trajectory data were provided by Beijing Advanced Innovation Centre for Big Data and Brain Computing (BCBD) (BCBD, 2020). The BCBD has applied natural language processing and other methods to extract information from public resources on 4626 confirmed patients from January 21 to February 20, 2020, including their gender, age, occupation, city of permanent residence, Wuhan/Hubei contact history, and patient trajectory (e.g., time, location, transportation, and event) (Fig. 1).

### Data pre-processing

The patient trajectories published by BCBD were inconsistent in terms of their formatting and details, which may be attributed to the various privacy concerns across different regions in China. Some patients were recorded at a fine spatial resolution. For instance, in Hainan province, data were available at the level of individual buildings, while the data in Beijing were relatively coarser. Therefore, the original data need to be processed and filtered.

Here, we aggregated all recorded locations of activities at the city level and used the city level as nodes for patients’ trajectories. The reason for this is threefold. First, mainland China has a top-down hierarchy, and each village and town can find the city to which it belongs. Second, the transportation system in China also has a hierarchical structure, similar to the urban system (Siwiak, Szczesny, & Siwiak, 2020), and movement between towns uses cities as intermediaries. Third, the city is the regional centre that is responsible for the treatment and tracking of confirmed cases on all lands under the jurisdiction of the city, including town and villages. In total, we have data on patients’ trajectories through 304 cities in China.

However, the patients diagnosed in Beijing and Shanghai only had recorded activity on the day of diagnosis; thus, the confirmed case data in these two cities are not included here. We believe that the elimination of these two cities will not affect the overall analysis and conclusions of our study. Therefore, the travel trajectories of 4051 patients in 302 cities were eventually covered in our analysis in the case study, discussed in Section 4. This means that we have the data of confirmed cases in 25 provincial-level units,^1^ including 20 provinces (excluding Qinghai, Hubei, Taiwan), 3 autonomous regions (excluding Xinjiang Uygur, Xizang), and 2 municipalities (excluding Beijing, Shanghai). It should be noted that the patients’ trajectory may cover the entirety of mainland China before they were confirmed as having COVID-19 in these 25 units.

**Fig. 2:**
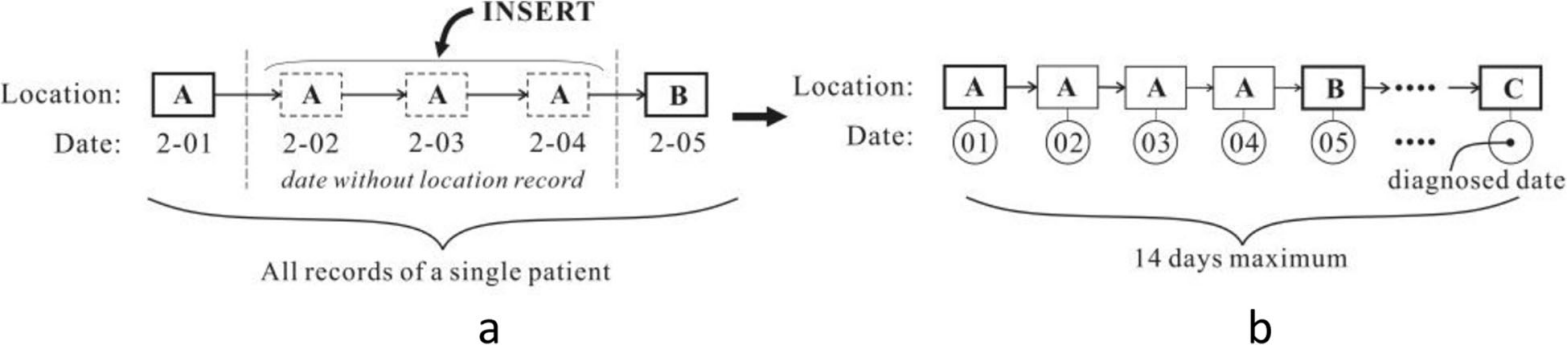
Completing the 14-day trajectories: **a** original trajectory data structure; **b** inserting missing dates between discontinuous dates

Moreover, in the BCBD data the trajectory information is recorded as the dates (2–01 or 2–05) with corresponding locations (A or B), as shown in Fig. 2a. There are no location data between two discontinuous dates. Here, we use the location of the previous date as a proxy for the location between dates. This idea has been used to complement the 14-day trajectories from the date the case is confirmed (here, 14 days is used as the virus incubation period), as shown in Fig. 2b. Given that the patients’ trajectories were re-constructed mostly by interviewing the confirmed patients, there is a slight chance that the records may not be fully accurate. We consider that this error is acceptable for our analysis, and discussion of the impact of this error is beyond the scope of this paper. We also assume the patient has visited no other cities between two discontinuous dates, because the places/cities and dates of visitation should have been recorded if other places/cities were visited.

**Fig. 3:**
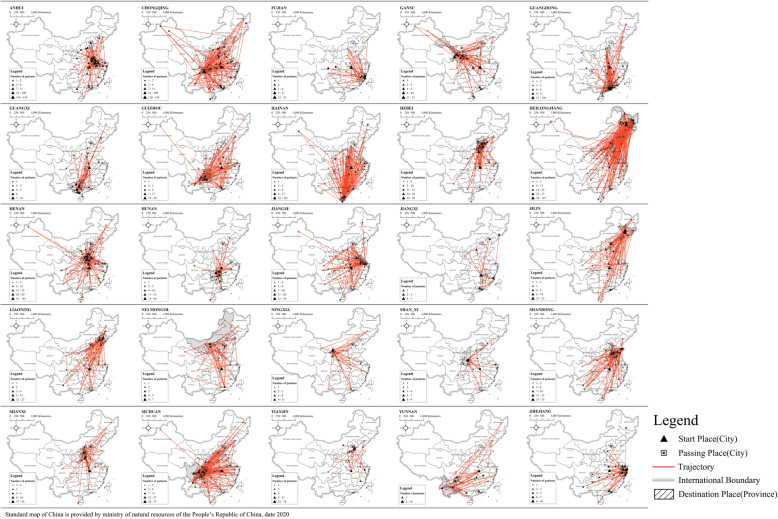
Trajectories of the confirmed patients in cities of 25 provincial-level units

In this way, we generated a continuous spatiotemporal trajectory for each of the patients in the 14-day incubation period at the city level, as shown in Fig. 3. Observing the distribution pattern of the activity trajectory in Fig. 3, we can see that each provincial unit presents a central radial pattern, with the confirmed province as the core, and there are many trajectory points in neighbouring provinces. This indicates that a considerable number of patients visited other cities/places before they were diagnosed and confirmed in a specific city/place during their incubation periods. Conventional analysis of the human mobility related to the transmission of COVID-19 focuses on where the patient was finally diagnosed. The truth is that not all patients stayed in the same places before they were diagnosed, as shown in Fig. 3, and the city they passed through might be the actual location of their infection. However, these locations are ignored in existing research, which will conceal the actual spatial spread of the epidemic. Therefore, it is necessary to analyse patients’ movement patterns and paths to understand this further, which is one of the aims of this work.

**Fig. 4:**
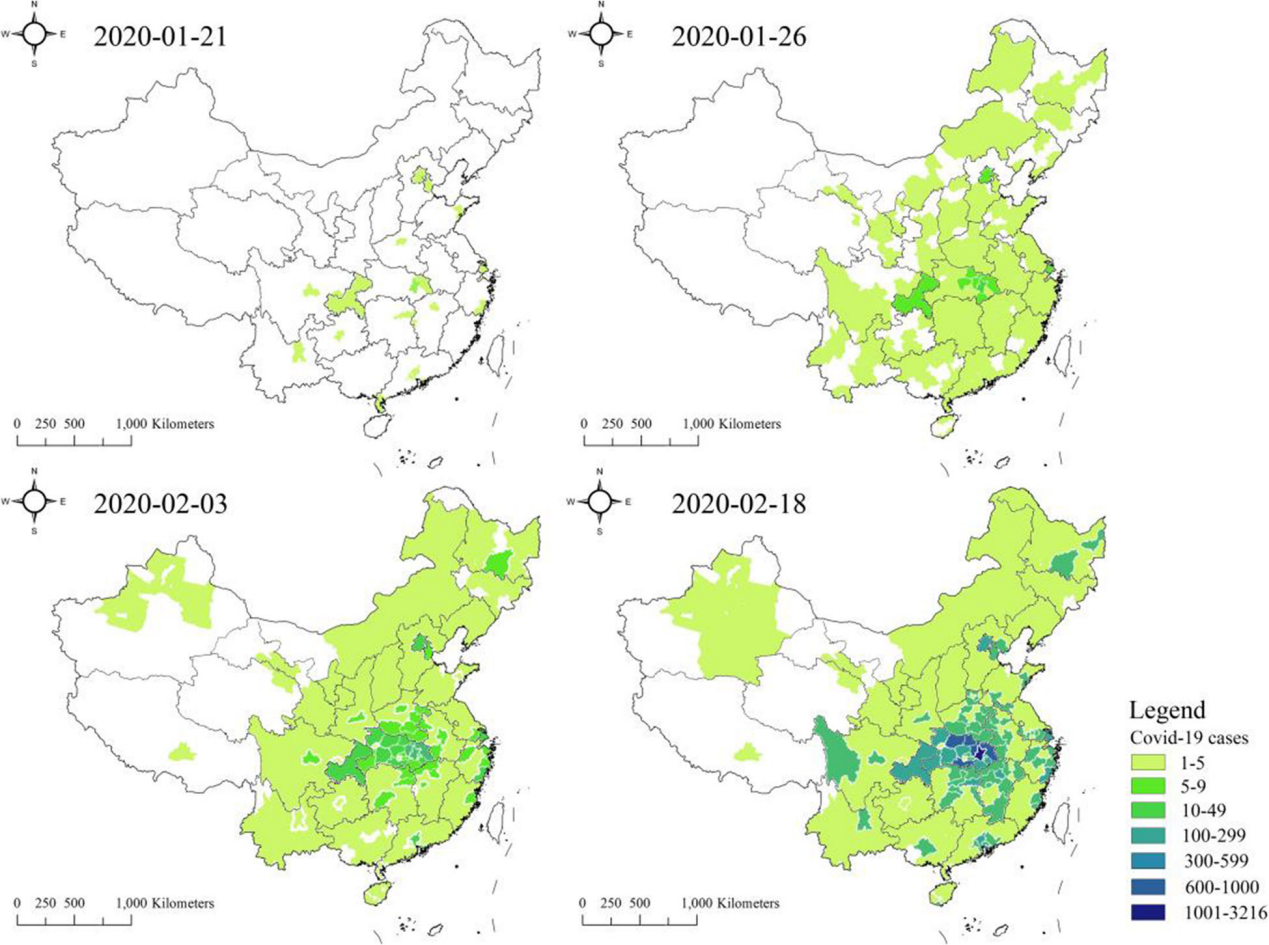
Number of confirmed patients accumulated in cities in 4 stages

Data concerning the cumulative number of cases and the daily increase in the number of confirmed cases are released by the national and provincial health commission in China (see Fig. 4). Figure 4 shows the development of the number of confirmed patients in each city, which should be compared with Figs. 6b and 8c.

## Methodology

This work proposes three steps to analyse patients’ movement and case data to gain a deeper insight into COVID-19 transmission to prevent its further spread. We first analyse the mobility and travel patterns of patients in cities during the incubation period alongside the case data from both spatial and temporal perspectives. Then, we extract the space-time hotspots/clusters of patient incubations to understand the dynamics of movement and transmission over space and time. Finally, we detect the order and stage of transmission within cities based upon the case data. Using patients’ mobility patterns and the order of case transmission, the spatiotemporal stages and patterns of transmission (local or cross-regional) can be drawn.

### Travel patterns vs. transmission—local or regional outbreak

As COVID-19 has a strong transmission capacity, cities where patients have stayed before their confirmation may have a high risk of virus transmission. Therefore, we will analyse the travel behaviour of patients in other cities before they are confirmed. First, the patients who have been to Wuhan city and Hubei Province will be identified to understand the initial transmission from Wuhan to other cities. Then, the travel distance, daily visiting volume and length of stay of all the patients in a city will be analysed to help us understand patients’ mobility vs. virus transmission at the city level. This can help us to identify the key factors leading to the daily confirmed and accumulated cases in cities. Based upon patients’ travel distance and length of stay, it is possible to identify whether outbreaks in a city are local or part of the pattern of regional transmission. Based upon the numbers of daily visitors and lengths of stay, we can identify the cities which have been highly exposed to the virus even if their confirmed cases may not be high at that moment.

### Space-time paths of patients—space-time hotspots

The analyses above give perspectives in either space or time. Given that the viral transmission is continuously moving in space and time, it is better to understand the dynamic viral transmission through space–time paths built upon the trajectory points of all patients. The space–time path will be able to show areas with high concentrations of patients in space–time—namely, the high-risk clusters (e.g., cities or regions) (Siwiak et al., 2020). This fine spatiotemporal scale will reveal transmission patterns that might have been ignored in existing research based on the whole population’s mobility. This will help us to understand the stages and orders of transmission.

3D Kernel Density (3D-KDE) is a method that is widely used to simplify trajectories and display active trajectories in a volumetric manner with three-dimensional space-time (Andrienko & Andrienko, 2013). 3D-KDE was designed by adding a time dimension to the 2D kernel density (Nakaya & Yano, 2010). In order to simplify the computing process, the intermediate process of the paths will be ignored. 3D-KDE only calculates the spatial density of the start or endpoints at each moment (Amini et al., 2015). In this study, 3D-KDE employs a Gaussian kernel density algorithm. The spatiotemporal cube includes 50 temporal planes (the BCBD dataset covers 50 days), and each plane has been divided into 10 km grids. After computing, 600*400*50 three-dimensional matrices of spatiotemporal density can be obtained, and each point has a density value. The heat map is used to visualise the results of the density analysis. In this paper, *Voxler* was used to directly produce a continuous and smooth spatiotemporal density cube (Demšar & Virrantaus, 2010). The equation for 3D-KDE is as defined in Eq. 1 (Brunsdon, Corcoran, & Higgs, 2007):

1 $$ f\left(x,y,t\right)=\frac{1}{n{b}_s^2{b}_t}{\sum}_i{K}_s\left(\frac{x-{x}_i}{b_s},\frac{y-{y}_i}{b_s}\right){K}_t\left(\frac{t-{t}_i}{b_t}\right), $$

where f (*x, y, t*) represents the kernel density at the position of *x, y, t*; *n* is the number of confirmed patients in each city on one plane; and *b*_*s*_ and *b*_*t*_ represent the search bandwidth in space and time, respectively.

### Lagging correlation between cities—case/virus spreading order

The step above focuses on exploring patients’ hotspots (before confirmation) and their dynamics in space–time. This step aims to explore whether the increasing number of patients in each city has a lagging correlation (Kang, Choi, Kim, & Choi, 2020). This means that the growth patterns of the two cities are similar but there is time lag, which could be used to analyse the order of spread of COVID-19. This can be used to further explain and associate the findings from Step 3.2 to build the orders and stages of the transmission, whether local or regional.

Sliding Window Time Lag Cross-Correlation (SWTLCC) is used in this research to achieve this goal, which originates from the combination of the methods of sliding window (SW) and time lag cross-correlation (TLCC). TLCC can identify directionality between two signals, such as a leader–follower relationship in which the leader initiates a response that is repeated by the follower. The sliding window can estimate the correlation between two sets of data at a smaller time granularity. According to the results of the sliding window, TLCC could be used to further detect which set of data changes caused a change in another set of data to determine the sequence of events (Podobnik, Wang, Horvatic, Grosse, & Stanley, 2010).

SWTLCC takes out fragments of the window size from the two sets of data and calculates the TLCC values of these two fragments. Then, as the window slides from front to back, the correlation of the entire set of data can be calculated. Compared with TLCC, SWTLCC can analyse whether there is a correlation between the two sets of data at a shorter time granularity and at what time the correlation between the two groups of data occurred.

We first offset one set of data and use the offset data to continuously analyse the degree of deviation between data of other cities and Wuhan to obtain the maximum correlation coefficient under the degree of offset. The degree of deviation can indicate the time sequence of the current city data affected by the Wuhan data, while the maximum correlation coefficient indicates whether the current city data and Wuhan data show similar trends.

## Results

Here, we present the exploratory analysis of the trajectory data from January 21 to February 20, 2019, when the outbreak of COVID-19 started in Wuhan and was transmitted to the rest of the country at its peak.

### Travel behaviours of the patients

#### Patients who have been to Hubei

**Fig. 5:**
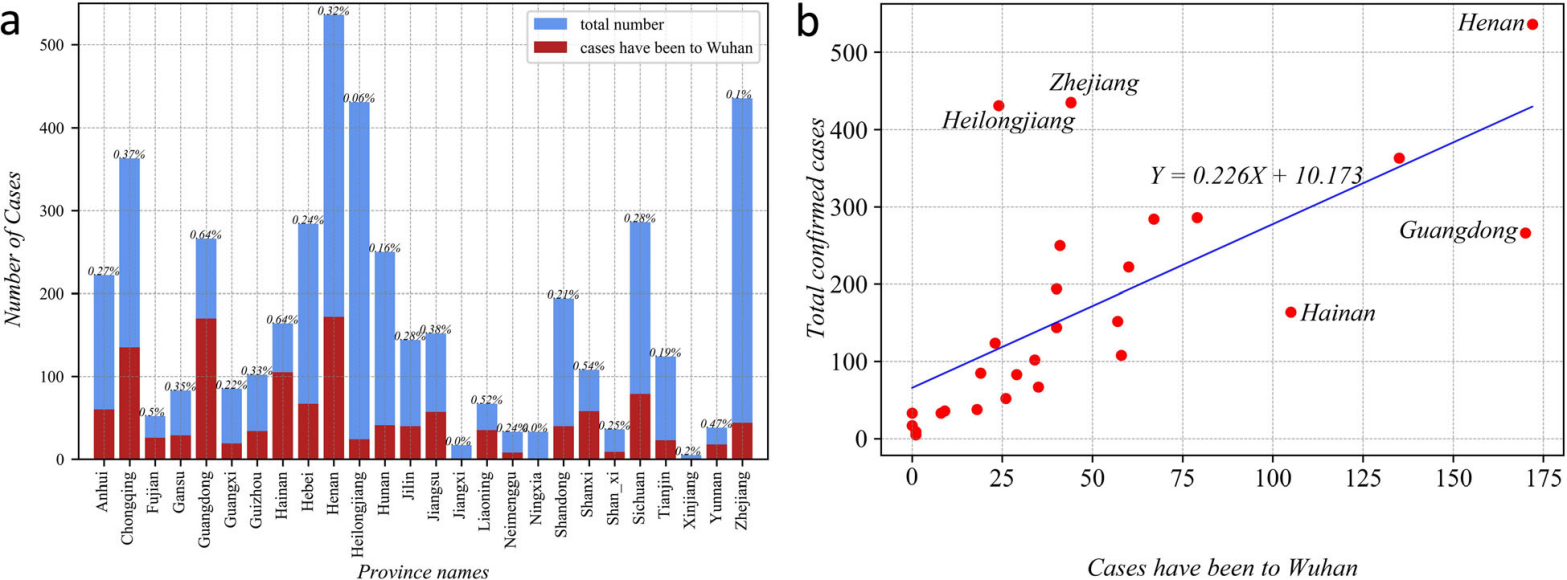
Number of cases that have travelled to Wuhan vs. total number of confirmed cases at the provincial level: **a** case numbers, **b** their correlation

Figure 5a presents the total number of confirmed cases and patients who have been to Wuhan (i.e., Hubei province), which varies significantly among the 25 provinces. For example, the numbers of cases in Zhejiang, Henan, Heilongjiang, Chongqing, Sichuan, and Guangdong are significantly higher than in other provinces. When the total number of confirmed patients is small, it strongly correlates with the number of patients who have been to Wuhan, as shown in Fig. 5b. Moreover, when the number of patients conspicuously increases, the proposed relationship is disturbed and shifted. For example, Zhejiang Province and Heilongjiang Province have many confirmed cases with a relatively low proportion of cases that have been to Hubei. The situation in Guangdong Province, however, is just the opposite. It has many cases that have been to Hubei, but the total number of confirmed cases did not rise sharply. This feature may reflect the difference in the spread mode of the virus between Hubei and other provinces. For example, the spread in Heilongjiang was caused by its famous local winter tourism activities. A small number of confirmed patients stayed there for a long time (local outbreak), which caused many local infections. in comparison, the outbreak in Zhejiang Province was due to many patients coming in from elsewhere.

#### Daily visit patients in cities

Since the trajectory data are detailed, it is possible to derive these patients’ daily distribution and obtain the total number of patients visiting each city every day. Here, we introduce the concept of “daily visit patients”, which represents the number of patients who were in their incubation period in each city every day (before being diagnosed). Additionally, if we add up the number of “daily visiting patients” in a period, we can find the “total number of visiting patients” in a particular city. In this part, we only consider those patients who have been to Wuhan/Hubei and returned, because the data focus on how infected people who have been to a high-risk zone can spread the virus to a larger area. Thus, this can reflect the spread pattern of COVID-19 better.

**Fig. 6:**
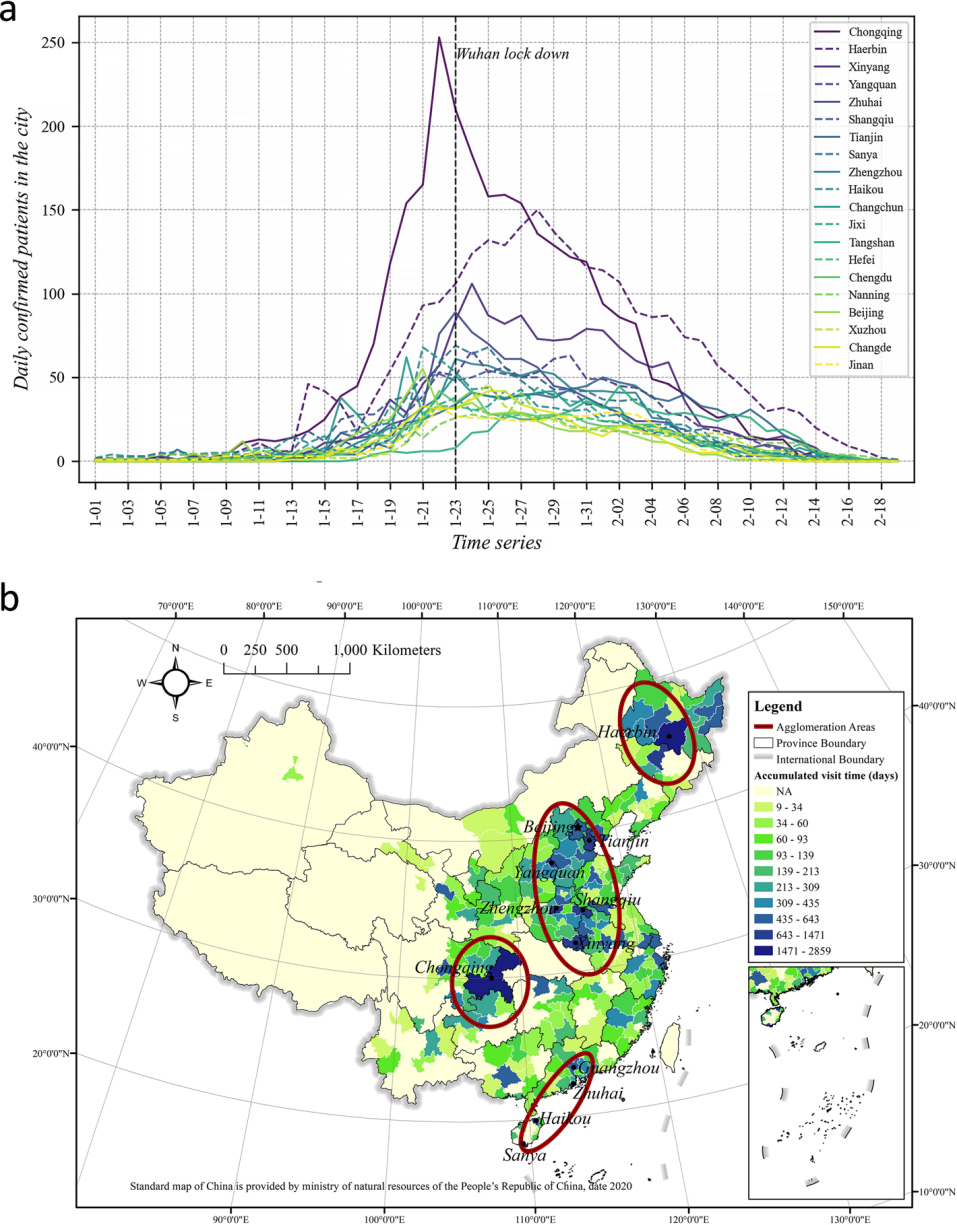
Number of patients (before diagnosis) in cities who have been to Hubei: **a** daily number of visiting patients; **b** accumulated number of visiting patients

Figure 6a shows the top 20 cities with the most patients visiting each day, growing rapidly in the first few days. However, after the lockdown of Wuhan, those numbers began to decline significantly, even though more patients were confirmed. Among others, Chongqing had a comparatively higher number and reached its peak first. Ha’erbin’s daily number of visitors was second only to Chongqing, but its growth rate seemed slower than that of other cities. The numbers of daily patients in Hunan Province (Xinyang, Shangqiu, Zhengzhou), Shanxi Province (Yangquan), Guangdong Province (Zhuhai), Hainan Province (Sanya, Haikou), and Tianjin are also relatively high.

By summing the time series data of each city, we derived the total number of patients visiting each day; this is shown in Fig. 6b. In terms of the overall pattern, four prominent agglomeration areas were formed: 1) patients returning from Hubei formed a relatively continuous cluster area between Hubei and Beijing, consisting of four provinces, including Anhui (Hefei), Shandong, Henan (Shangqiu), and Hebei; 2) Chongqing and Ha’erbin formed two distribution areas centred on themselves in the southeast and northeast, respectively; and 3) in the southern coastal areas, the Pearl River Delta and Hainan Province have formed another distribution area, reflecting the simultaneous existence of continuous transmission and cross-regional transmission.

#### Average movement distance of patients

As shown in Fig. 3 above, the movement trajectories of the confirmed patients in each province are quite variable. For example, Chongqing, Guangdong, Heilongjiang, and Sichuan have larger-scale activity trajectories, which is consistent with the fact that these provinces have more confirmed cases. In the Henan and Zhejiang provinces, on the contrary, the spatial distribution of the trajectories displayed does not match with the highest number of confirmed cases. Furthermore, there were not many confirmed cases in Hainan, though it has shown a large scale of activity trajectories.

**Fig. 7:**
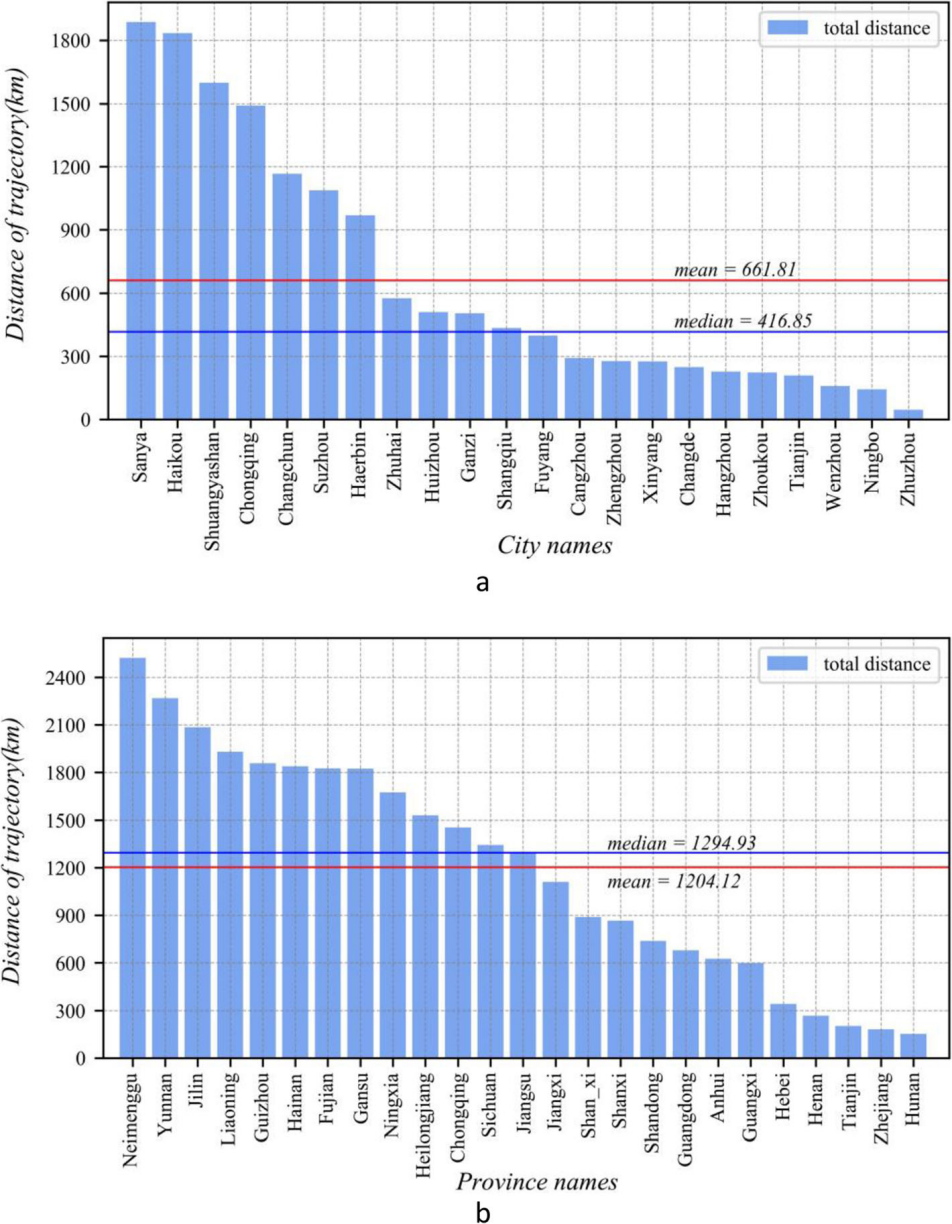
The average 14-day total travel distance of individual patients in **a** the top 22 cities and **b** 25 provincial-level units

To understand the seeming inconsistency of the activity trajectories with the number of confirmed cases, we simplified the distance that each patient moved (the Euclidean distance between the cities where the patient was for 14 days prior to being diagnosed and the city where they were confirmed to have COVID-19). Then, we calculated the average moving distance of each city and province (the total travel distance of patients in a city/province divided by the total number of patients in the city/province). The result is shown in Fig. 7.

At the city level, some cities far away from Wuhan, such as Sanya and Haikou (Hainan Province), as well as Shuangyashan, Changchun, and Harbin (Northeast China), have high moving distances, while Chongqing, which is very close to Wuhan, also has a high value.

At the province level, provinces with higher moving distances are generally farther from Hubei (except Chongqing), such as Inner Mongolia and Yunnan, as well as provinces in the northwest and northeast regions. This is well understood, indicating that patients who travelled from Hubei province at the beginning of the virus outbreak reached these provinces after moving a long distance. While Chongqing not only had many confirmed patients, these people also had moved across a large spatial distance before being diagnosed, which implies that there was a regional outbreak. On the contrary, Henan had the most confirmed patients, but its average regional migration distance is relatively small. Such results may indicate that the confirmed patients only moved within a small geographic area, implying that there was a local outbreak in Henan, similar to Zhejiang.

#### Staying time of patients

**Fig. 8:**
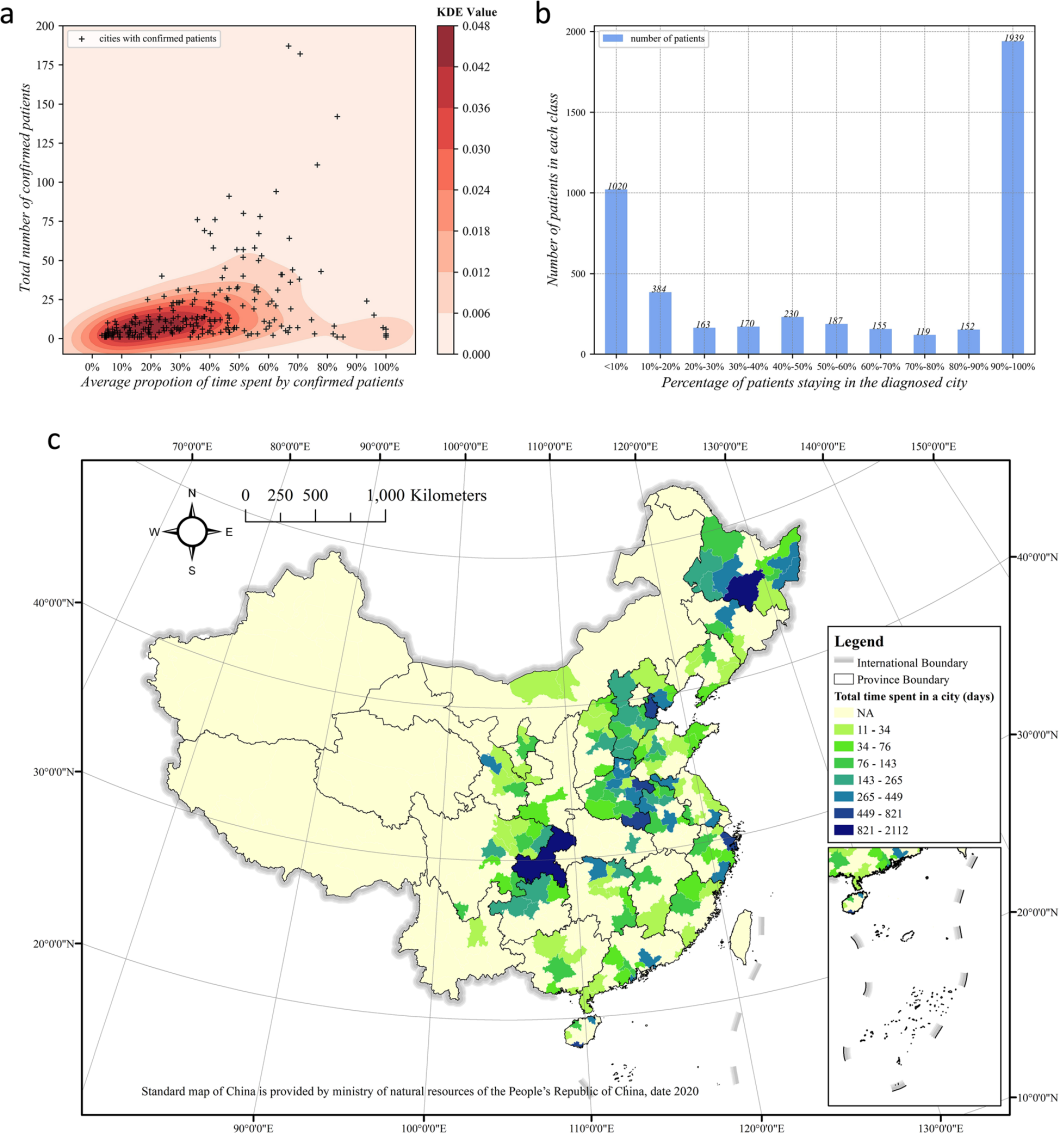
Length of stay of patients in cities: **a** the average proportion of length of stay vs. the total number of patients confirmed to have COVID-19; **b** the stay ratio of patients confirmed to have COVID-19 in the cities where they were diagnosed; **c** the total length of stay of patients

As discussed above, many patients visited other cities besides the city where they were confirmed to have COVID-19. Due to the strong transmission capacity of the virus, it is expected that the city where patients have stayed the longest will face the highest risk of virus transmission. Therefore, we analysed the length of time that the patients confirmed to have COVID-19 stayed in each city; the results are shown in Fig. 8.

Figure 8a shows a scatter plot of the average length of time stayed of all the patients in the city, divided by the total length of their trajectory and the total number of patients in each city. It shows that in more than half of the cities, the proportion of time spent by all the patients in this city is less than 50%. A reasonable assumption could be that patients were not infected with the virus in the city where they were confirmed to have COVID-19 but just visited there to work or seek medical treatment, while the city they passed through was the actual location of their infection. To confirm this, we converted the perspective to the patients by calculating the proportion of time each patient spent in the city where they were diagnosed (we name this value the “stay ratio”). We generated a data distribution diagram (reclassified at 10% intervals). Nearly 50% of patients spent more than 90% of their time in the city where they were confirmed to have COVID-19, while nearly 30% of patients had a stay ratio of less than 50%, as shown in Fig. 8b. From Fig. 8a,b, we can also see that if there were more patients in a city, there will be a higher probability that these patients never left or only left the city for a short period of time. Although we know that there are many such patients, there is still a considerable number of patients who did not spend most of their time before being diagnosed in the city where they were confirmed to have COVID-19 (low stay ratio).

Figure 8c shows the spatial distribution map of the average proportion of time stayed by patients in each city. Comparing Figs. 6band 8c, a remarkably similar pattern could be found—there are four key zones, as described in Section 4.1.3. Comparing these two figures with Fig. 4, the patterns are very similar to the final stage of the accumulative case patterns on February 18, 2020. This indicates that there is a strong association of patients’ mobility patterns with the virus transmission, which will be further explored in the following subsection.

#### Patients’ mobility vs. case numbers

As shown in Fig. 6a, about ten cities had significantly more daily visits by patients than other cities. Chongqing Municipality and Ha’erbin are particularly prominent, while most other cities are at the same level. Among these cities with higher numbers, provincial capital cities and municipalities are ranked lower, while second- and third-tier cities such as Xinyang, Yangquan, and Zhuhai are ranked higher, with more confirmed patients active in these cities every day. However, if we use the traditional number of confirmed patients (as shown in Fig. 5b) to rank cities, the larger provincial capital cities are ranked higher, such as Beijing, Chongqing Municipality, and Shanghai. Using these two methods, the ranking of the cities is vastly different, as shown in Table 1.

**Table 1:** The city rankings based upon the number of daily patient visits, daily confirmed patients (as of February 18, 2020), and daily stay time proportion

City ranking using daily patient (before confirmation) visits number (top 10)										
City	Chongqing Municipality	Ha’erbin	Xinyang	Yangquan	Zhuhai	Shangqiu	Tianjin	Sanya	Zhengzhou	Haikou
Count	2859	2614	1471	1011	972	936	933	933	869	867
City ranking using daily confirmed patient number (top 10)										
City	Chongqing Municipality	Ha’erbin	Wenzhou	Ningbo	Tianjin	Shangqiu	Zhuhai	Changde	Xinyang	Suzhou
Count	336	187	182	142	111	94	91	80	78	76
City ranking using daily stay time proportion (top 10)										
City	Ningbo	Tianjin	Wenzhou	Zhoukou	Ha’erbin	Shangqiu	Hangzhou	Xinyang	Zhengzhou	Chongqing Municipality
Proportion	83%	77%	71%	67%	67%	63%	58%	57%	57%	52%
Patient number	142	111	182	64	187	94	53	78	67	336

It can be noticed that cities with higher rankings using the first method are generally not ranked high when using the second method (except Chongqing Municipality). This means that, for example, in Xinyang and Shangqiu, many confirmed patients have visited here during the incubation period, but they did not end up staying there and were eventually diagnosed in other cities. Thus, there are two assumptions here: first, that these patients were infected in Xinyang or Shangqiu and then returned to other cities to be diagnosed; second, that these patients went to other cities for medical treatment after they developed symptoms, and they were eventually diagnosed in other cities. In either case, cities such as Xinyang are particularly important for disease prevention and control because these cities might be the actual places where the spread of the virus occurs. However, if we only use the data of patients confirmed to have COVID-19, we cannot discover the existence of these second- or third-tier cities which are at high risk (such as Sanya and Haikou).

Furthermore, the result shown in Fig. 6 is different from the existing literature, which used aggregated mobility flow derived from mobile phone data at prefectures in China (please refer to Jia et al., 2020, Fig. 1). Moreover, based upon the proportion of the length of stay of patients confirmed to have COVID-19 (Table 1), we can see that Tianjin, Ningbo, and Wenzhou (cities in Zhejiang); Zhoukou, Xinyang, Zhengzhou, and Shangqiu (cities in Henan); and Ha’erbin all have very high rates of patients staying. This indicates that the patients confirmed to have COVID-19 in these cities have indeed stayed in these places for a long time, which is consistent with the conjecture in Section 4.1.3—that is, these areas are likely to have local outbreaks.

### Space-time paths of patients after returning from Hubei province

**Fig. 9:**
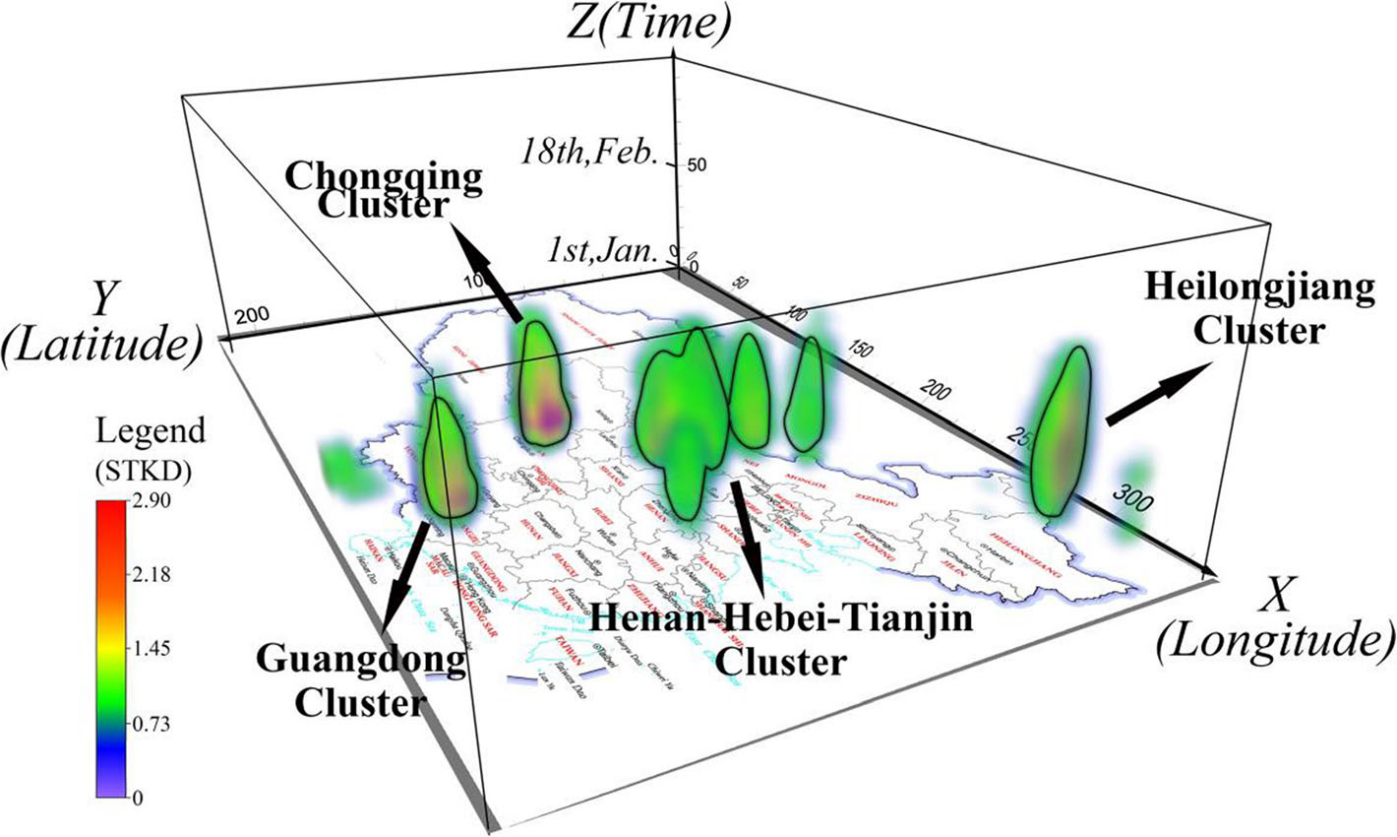
Space–time paths clusters of patients after returning from Hubei Province

Section 4.1 analyses the mobility of the patients from either spatial or temporal perspectives. In this section, the spatial and temporal dimensions are integrated to understand the transmission dynamics in integrated space–time. We hereby specifically extract the spatiotemporal records of the patients who have been to Wuhan/Hubei before, then plot their paths after returning from Hubei. After calculation and rendering, as described in Section 3.2, the result is obtained and shown in Fig. 9. The three coordinate axes represent two-dimensional space (i.e., X and Y) and one-dimensional time (i.e., Z). The space–time density graph has become a columnar shape unevenly clustered in some places. Four high-density agglomeration areas were identified and named according to their spatial distributions (see Fig. 9), including the Heilongjiang cluster, Chongqing cluster, Henan–Hebei–Tianjin cluster, and Guangdong cluster.

The distribution of spatial activity density of patients shows a similar pattern as that of Fig. 6b in Section 4.1.1 and Fig. 8c in Section 4.1.3. A spatial circle is formed around Hubei Province, whilst some provinces, including Chongqing and Henan surrounding Hubei Province, show high-density agglomeration. Similarly, Guangdong, Hainan, and northeast China, which are far from Hubei, also show high-density agglomeration.

**Fig. 10:**
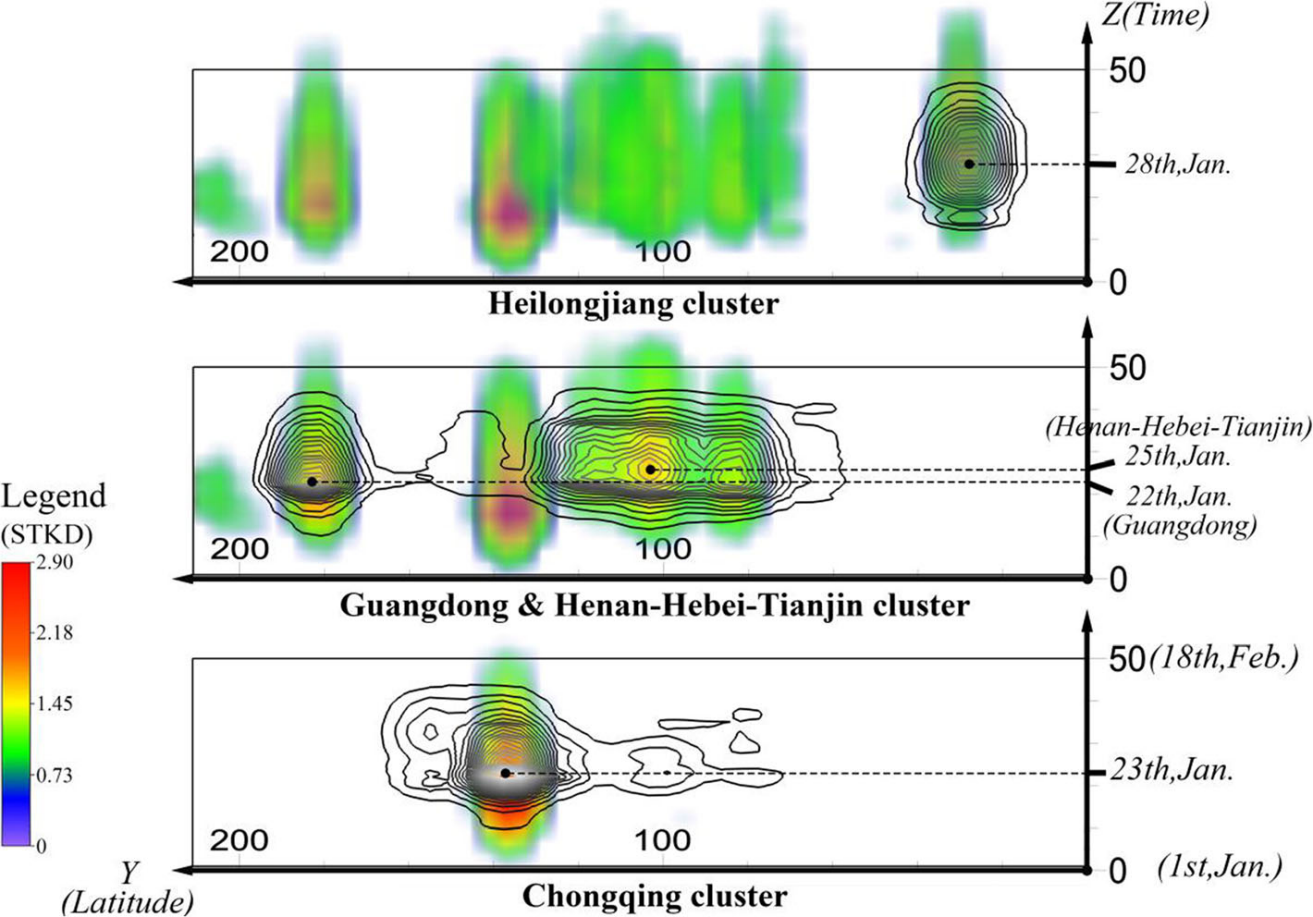
Temporal sections of the space–time cube

Observing the space–time cube from the side and generating a gradient density curve, we can observe similar results as shown in Fig. 10. The X-axis represents the profile along the latitude direction, and the Y-axis is the tempol line running through 50 days. This shows that Chongqing has the highest cluster density and the most prolonged duration, with a remarkably high density from about early January to the end of February (almost 50 days). While Guangdong and Henan-Hebei-Tianjin cluster were also formed early, the Heilongjiang cluster was formed a few days later than the other clusters. There is a clear sequence of the time when patients start to gather in different clusters. The cluster in Guangdong Province reached its peak earliest on January 22, and the cluster in Chongqing reached its peak on January 23. The clusters in these two regions nearly disappeared at the end of the time range included in the data (February 18), which means that the confirmed patient stopped activities in these two areas within 1 month. The Henan–Hebei–Tianjin cluster peak appeared later, on January 25, while the Heilongjiang cluster appeared latest on January 28. These two clusters showed no signs of disappearing before February 18, indicating the patient activities of these two clusters lasted longer, exceeding the time frame of our study. It is speculated that the virus transmission may have stages due to stages of patient movements revealed here, which will be explored in the following subsection.

### Orders and stages of virus transmission

#### Orders of virus transmission

As observed from Fig. 6a, the daily increase in the number of patients in each city seems to have a chronological order. Wuhan grew rapidly, and Chongqing’s data grew with Wuhan’s data, while other cities grew more slowly. The sequence of data changes may represent the order of diffusion in time. Figures 9 and 10 collectively further illustrate that there is order and possible cross-regional transmission of the virus. To explore this further, we apply the SWTLCC analysis introduced in Section 3.3 to validate the associations between the mobility with the virus transmission.

**Fig. 11:**
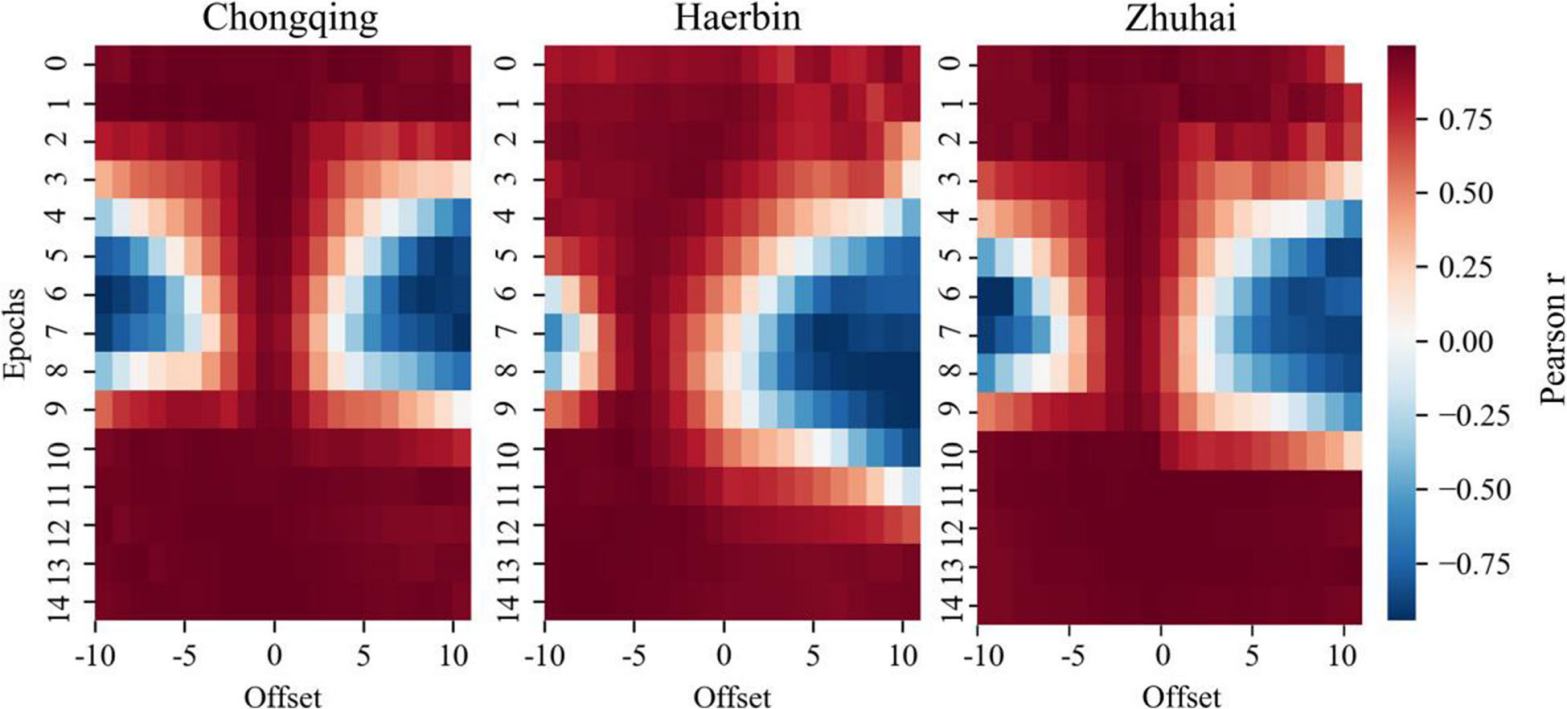
Data offsets of Chongqing, Ha’erbin, and Xinyang (WTLCC)

We first use the data of Wuhan as the benchmark data to perform SWTLCC with a window size of 20 (approximately half of the total date recorded with patient activity tracking, to ensure that there are enough data in the window). The data of all other cities are compared with the data of Wuhan, and the result is shown in Fig. 11. Selecting the analysis results of the top three cities other than Wuhan, the data offset in Chongqing is not obvious, while the data in Ha’erbin and Zhuhai have a noticeable offset to the left in the middle part, this indicates that the data of Wuhan are leading the data of these two cities to change. Moreover, the change patterns of the rest cities in Fig. 6a are similar to those of Ha’erbin or Zhuhai, strongly guided by Wuhan data.

**Fig. 12:**
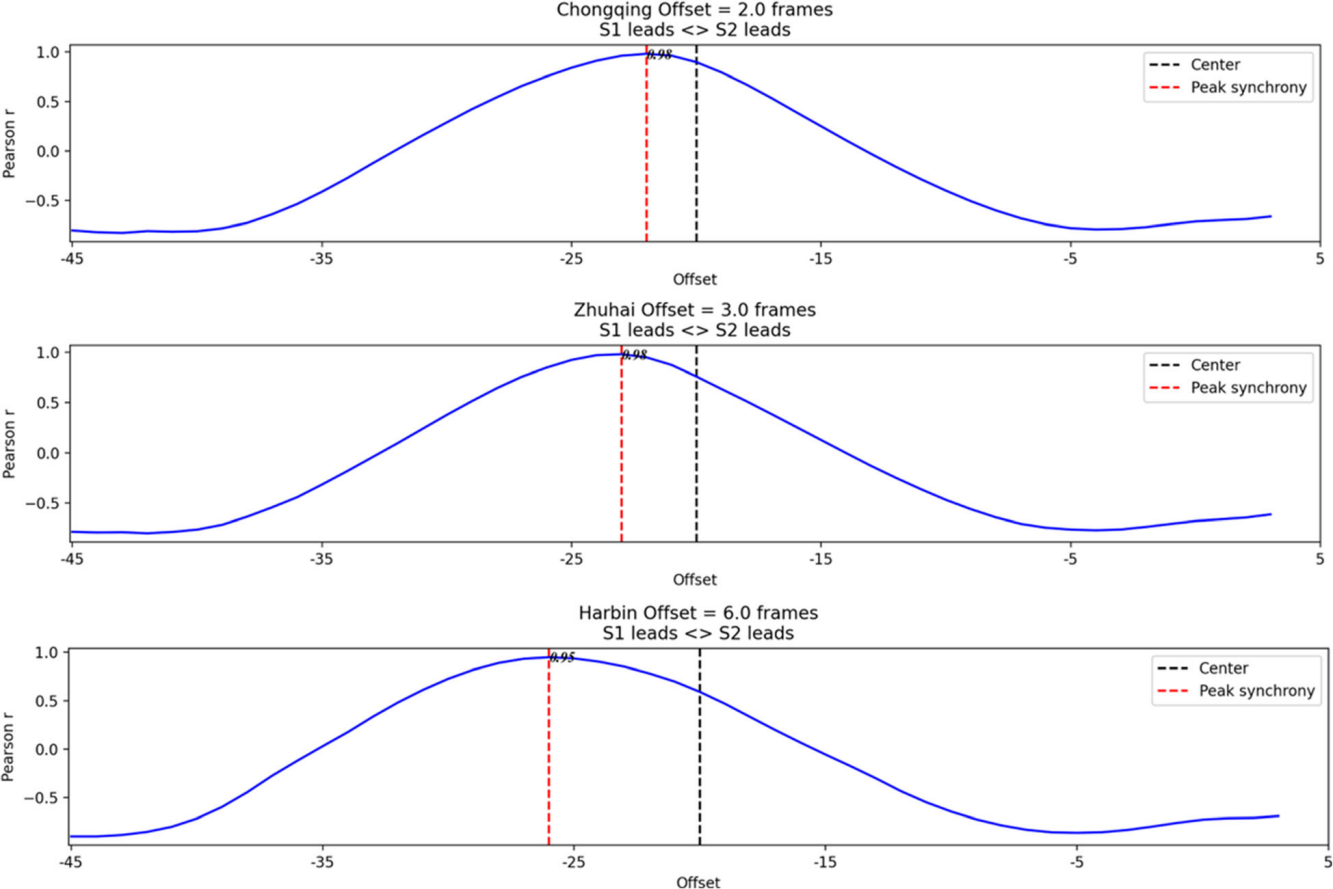
Different offset values of three cities data (TLCC)

According to the method described in Section 3.3, we apply the TLCC method to find the data sequence. After calculating, the data of three representative cities are selected, as shown in Fig. 12 below. The results of the TLCC analysis for the data of Chongqing, Ha’erbin, and Zhuhai show that the maximum correlation coefficients that these three cities can achieve are all above 0.9, but the offsets increase sequentially. Chongqing has the most minor offset; Wuhan’s data began to change immediately after it began to change, while Zhuhai was left behind a little, and Ha’erbin’s data only began to change after six shifts. This shows that the three cities are affected by the spatial activities of Wuhan patients in a sequence.

**Fig. 13:**
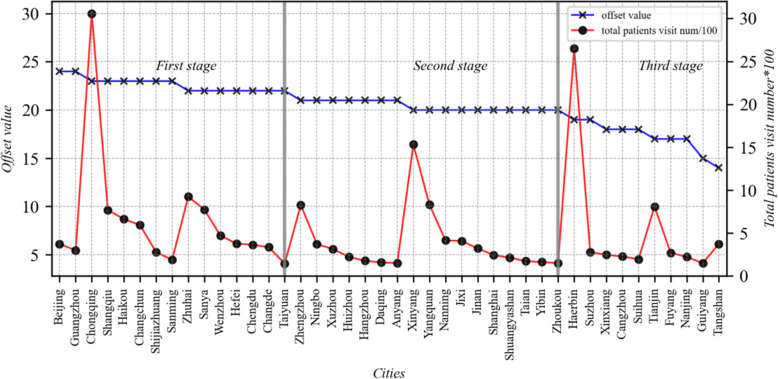
Joint graph of the total number of patient visits, data offset, and maximum correlation coefficient in each city

In order to visualise all the data, it is necessary to filter the data of other cities. First, the correlation coefficient must be greater than 0.9 (high relevant cities). Secondly, the number of visits by confirmed patients in a city needs to reach a certain number. Through the observation of the data, the value of 400 is selected as the screening criterion; finally, the cities with a positive offset are selected, which means these cities are affected by the Wuhan data. After screening, the result is shown in Fig. 13. The blue curve is the reverse offset value and is sorted. The larger the value, the faster the data of the city were affected by the Wuhan data.

According to the observation of the offset value curve in Fig. 13, we divided the sequence of these cities affected by Wuhan data into three stages. The offset is within 3 as the first stage, 3 to 5 offsets as the second stage, and more than 5 offsets as the third stage. In each stage, there are some prominent cities. In the first stage, the data of Beijing and Guangzhou change fastest, almost synchronised with the data of Wuhan. Chongqing also changes very quickly, and its total number of patient visits is extremely high, which means that the city has the fastest response and the largest number of patients. In the second stage, Zhengzhou, Xinyang, and Yangquan had a higher number of patient visits in this stage; in the third stage, Ha’erbin and Tianjin have higher values, while Ha’erbin is particularly high. The concentration of confirmed patients in these two cities was only after the epidemic in Wuhan developed for a period, but the local virus spread fast, which led to the number of confirmed patients rising rapidly.

#### Stages of COVID-19 transmissions

**Fig. 14:**
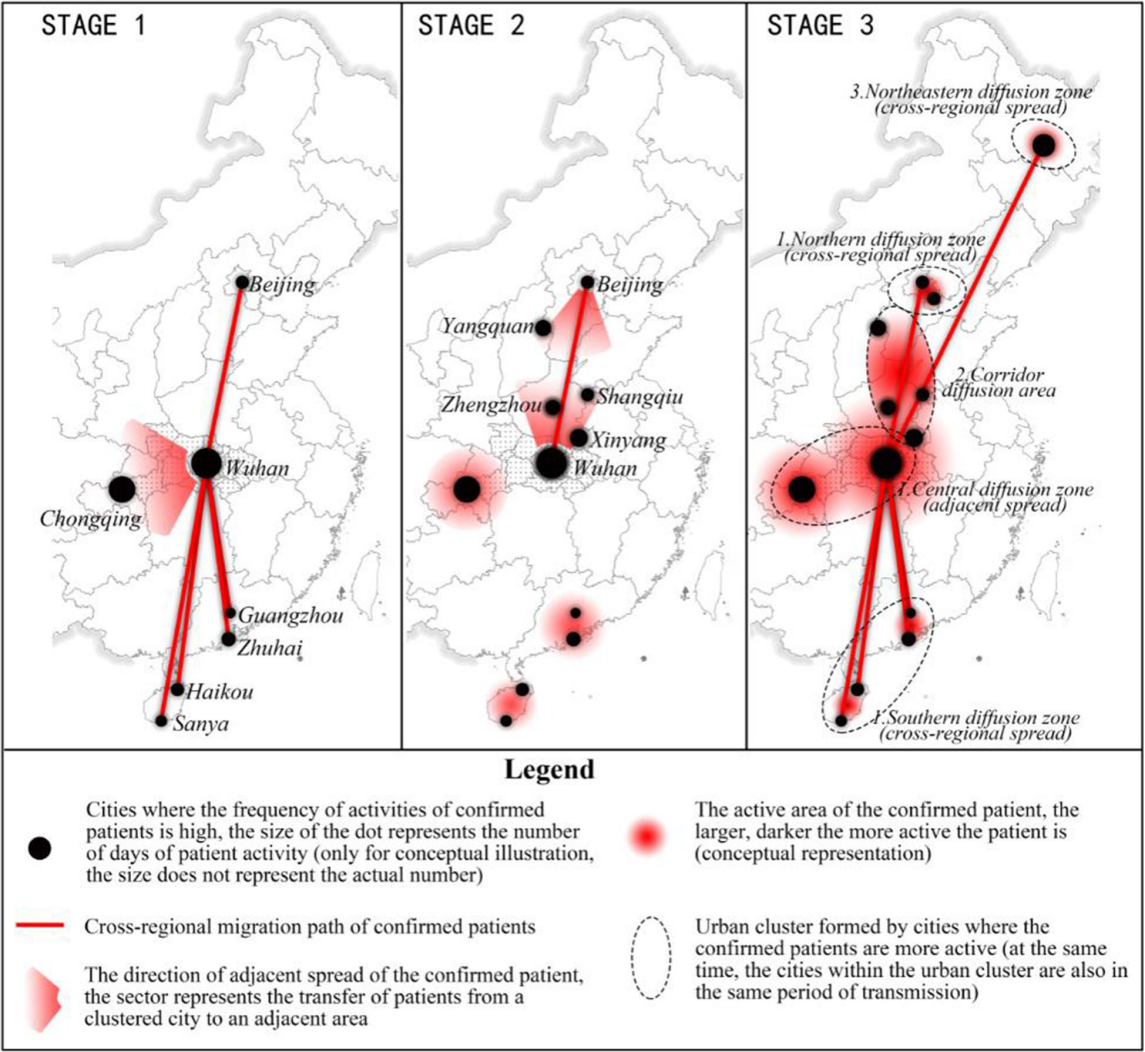
Three key stages of COVID-19 transmission in mainland China

Based upon the analysis above, we can now draw the development stages of COVID-19 transmission in mainland China as shown in Fig. 14.

In the first stage, the confirmed patients in Wuhan increased rapidly and spread out from Wuhan as the centre, first to Beijing, Guangzhou, Zhuhai, Haikou, and Sanya in a cross-regional manner, as well as to Chongqing in an adjacent diffusion way. This may be achieved by frequent transport connections between these cities with Wuhan via train or air flights.

In the second stage, a corridor zone was formed in the plain urban area between Beijing and Wuhan, where the spatial activities of the confirmed patients spread. According to the approach of spreading, the number of patients visited Xinyang, Shangqiu, Yangquan, and Zhengzhou increased rapidly. This more like the local spreading via train or other transport modes.

In the third stage, the patients spread to Ha’erbin and Tianjin in the form of cross-regional or adjacent spreading through some special events. At this stage, two cross-regional spreading zones in the south and the north were formed, the adjacent spreading zone and the corridor spreading zone formed in the central area, and the cross-regional diffusion zone formed in the northeast.

According to Fig. 6, very few confirmed patients in Heilongjiang (Northeast diffusion zone), Hebei, and Henan provinces (Corridor diffusion zone) have been to Hubei. This implies that these provinces have local outbreaks, while Guangdong and Chongqing, on the contrary, have become regional new sources of virus spread.

After three stages, two different types of transmission areas were formed. One is the adjacent diffusion area closer to Hubei, such as Chongqing, Henan, and Hebei, and the other is the cross-regional diffusion area that is farther away from Hubei, such as Heilongjiang, Guangdong, and Hainan.

According to the results in Fig. 13, adjacent diffusion occurs in the second stage (except Chongqing) and the mechanism is the gradual spread of patients in a connected area, just like the corridor area shown in Fig. 14. On the other hand, there are two different generation mechanisms for cross-regional transmission. The transmission in Guangdong and Hainan is mainly caused by migrants and it occurs in the first stage; these two provinces and Hubei have a relatively close relationship during the Spring Festival. However, the spread in Heilongjiang is caused by special tourism events, so its spread occurs in the third stage.

Table 2 summarises the virus transmission stages and types of diffusion based upon the patients’ mobility patterns. Here, the adjacent diffusion means that the cases are spreading at the local or regional level, and those cities are the focus cities in such spreading. The cross-regional diffusion means that the spreading is cross-regional, though the spread in those cities at stage 3 is more like a local outbreak in Ha’erbin and Tianjin.

**Table 2:** The development stage of adjacent diffusion and trans-regional diffusion

Stage	TYPE of diffusion	
	Adjacent diffusion	Cross-regional diffusion
Stage 1	Chongqing	Beijing, Guangzhou, Haikou, Zhuhai, Sanya
Stage 2	Zhengzhou, Xinyang, Yangquan, Shangqiu	
Stage 3		Ha’erbin, Tianjin

## Discussion, summary and conclusions

The COVID-19 pandemic has had a significant impact on global social and economic activities. As mainland China was the region where the early outbreak started, studying the transmission of COVID-19 is of great significance to formulating effective epidemic prevention and control measures. By using patient trajectory data, this study has discovered some new insights into the spatial pattern of disease transmission from the following perspectives:

### Flaws in using the number of confirmed patients in existing studies

The result of Section 4.1 indicates that during the 14-day incubation period, patients can carry out a wide range of activities across space and have opportunities to spread the virus to a larger area, not just the city where they were confirmed to have COVID-19. In this case, traditional data (cumulative number of confirmed patients in each city) only describes the final location of each patient and ignores the process of spatial movement—that is, it is static and cannot fully represent the real distribution pattern. Furthermore, Section 4.1 also shows that many patients did not carry out spatial activities in their confirmed city but were merely diagnosed in these cities. Thus, using the number of confirmed cases in each city as the basis to study the diffusion pattern data will cause errors.

### New results brought out by proposed spatiotemporal trajectory data analysis

Studying the spread of the virus by using individual patients’ trajectory data has yielded different results. By observing the daily number of patients visiting each city, we found that the number of patients in some cities could be remarkably high, even though the total number of confirmed patients in those cities was relatively low (Section 4.1.2). Some cities located in the southern region, such as Zhuhai, Sanya, and Haikou, and in the northern region, such as Shuangyashan, Changchun, and Harbin, are the main cities where the confirmed patients have the greatest mobility during the spread of the pandemic.

However, these cities are not first-tier cities with a large population. This will cause the effects mentioned in Section 4.1.4. Many confirmed patients visited relatively small cities during their incubation period (e.g., Xinyang, Yangquan, Sanya, and Haikou.), stayed there for a long time, and then returned to other cities. Whether these patients were infected in these small cities or not, they are more likely to be infected or infect other citizens when staying in these small cities. Therefore, these smaller cities may be where the infection and spread actually occur, and they seem to be ignored by traditional data.

The results in Section 4.1 show that four prominent areas of high-intensity patient activity have formed, and Section 4.2 shows that these areas are formed in three stages, which is further validated in Section 4.3 (Table 2). There is one contiguous diffusion area around Wuhan and Chongqing (stage 1), one corridor diffusion area between Wuhan and Beijing composed of some small and medium-sized cities (stage 1; Stage 2), and two cross-regional diffusion areas in the Pearl River Delta Area (Stage 2) and Heilongjiang (Stage 3). This corridor diffusion area is interesting; although the number of confirmed patients in these cities is not large, this corridor area covers a considerable part of northern China, from Beijing to Wuhan (Section 4.1.2). This means that the movement of confirmed patients in this area is relatively free, and eventually these confirmed patients will move to larger central cities, which might make the number of confirmed patients in these small and medium-sized cities appear quite small. This phenomenon is worth noting, because if the spread of the virus can be detected and controlled as early as possible the pressure on management and medical systems will be much reduced.

Furthermore, the use of patient trajectory data can also determine whether the confirmed patient has a phased spread of time and space. The number of patients visiting big cities such as Beijing, Guangzhou, and Chongqing started to increase at the earliest timepoint, while cities in the corridor area generally showed increases in the second stage. This shows that patients confirmed to have COVID-19 first spread the disease between large cities through cross-regional transmission and then infiltrate the smaller cities sandwiched between large cities. In light of this, we suggest that putting Wuhan into lockdown did make an important contribution to containing the spread of the virus.

### Limitation of this research

Although the use of patient trajectory data can describe the activities of patients confirmed to have COVID-19 on a finer spatial and time scale, we still cannot know where and when people were infected. Moreover, due to our data limitations, this study did not include the modes of transportation used by patients to move between cities, and it was also impossible to determine what activities the confirmed patients performed in the city, which will lead to a certain degree of error in the study, because the nature of activities carried out will significantly affect people’s chance of spreading the virus to others. One potential means to overcome this limitation would be to use the GPS trajectories of individual patients, subject to ethical standards and the individual patient’s approval, or the use of trace and tracking data, if available.

## Data Availability

Data used in this research is available at: https://github.com/BDBC-KG-NLP/COVID-19-tracker. The main part of this analysis and visualisations were partially conducted by using MATLAB and ArcMap.

## Footnotes

1 China’s administrative division system divides the country into 34 provincial administrative units at the first level, including 23 provinces (Anhui, Fujian, Gansu, Guangdong, Guizhou, Hainan, Hebei, Heilongjiang, Henan, Hubei, Hunan, Jiangsu, Jiangxi, Jilin, Liaoning, Qinghai, Shan_xi, Shandong, Shanxi, Sichuan, Yunnan, Zhejiang, Taiwan), five autonomous regions (Inner Mongolia, Ningxia, Guangxi, Xinjiang Uygur, Tibet), four municipalities (Beijing, Chongqing, Shanghai, Tianjin), and two special administrative regions (Macau, Hong Kong).

## References

[CR1] Amini, F., Rufiange, S., Hossain, Z., Ventura, Q., Irani, P., & McGuffin, M. J. (2015). The impact of interactivity on comprehending 2D and 3D visualisations of movement data. *IEEE Transactions on Visualization and Computer Graphics, 21*(1), 122–135. 10.1109/TVCG.2014.2329308. 26357026 10.1109/TVCG.2014.2329308

[CR2] Andrienko, N., & Andrienko, G. (2013). Visual analytics of movement: An overview of methods, tools and procedures. *Information Visualization*. London, England: SAGE Publications*, 12*(1), 3–24. 10.1177/1473871612457601.

[CR3] Backer, J. A., Klinkenberg, D., & Wallinga, J. (2020). Incubation period of 2019 novel coronavirus (2019- nCoV) infections among travellers from Wuhan, China, January 20 28 2020. *Eurosurveillance*. Sweden: European Centre for Disease Control and Prevention (ECDC)*, 25*(5). 10.2807/1560-7917.ES.2020.25.5.2000062. 10.2807/1560-7917.ES.2020.25.5.2000062 PMC7014672 32046819

[CR4] Badr, H. S., Du, H., Marshall, M., Dong, E., Squire, M. M., & Gardner, L. M. (2020). Association between mobility patterns and COVID-19 transmission in the USA: A mathematical modelling study. *The Lancet Infectious Diseases, 20*(11), 1247–1254. 10.1016/S1473-3099(20)30553-3. 32621869 10.1016/S1473-3099(20)30553-3 PMC7329287

[CR5] Bai, Y., Yao, L., Wei, T., Tian, F., Jin, D. Y., Chen, L., & Wang, M. (2020). Presumed asymptomatic carrier transmission of COVID-19. *JAMA - Journal of the American Medical Association*. United States: American Medical Association*, 323*(14), 1406–1407. 10.1001/jama.2020.2565. 32083643 10.1001/jama.2020.2565 PMC7042844

[CR6] Balcan, D., Colizza, V., Gonçalves, B., Hud, H., Ramasco, J. J., & Vespignani, A. (2009). Multiscale mobility networks and the spatial spreading of infectious diseases. *Proceedings of the National Academy of Sciences of the United States of America, 106*(51), 21484–21489. 10.1073/pnas.0906910106. 20018697 10.1073/pnas.0906910106 PMC2793313

[CR7] Balzotti, C., Bragagnini, A., Briani, M., & Cristiani, E. (2018). Understanding human mobility flows from aggregated mobile phone data⁎. *IFAC-PapersOnLine, 51*(9), 25–30. 10.1016/j.ifacol.2018.07.005.

[CR8] BCBD. (2020). *COVID-19-tracker*. Github https://github.com/BDBC-KG-NLP/COVID-19-tracker.

[CR9] Brunsdon, C., Corcoran, J., & Higgs, G. (2007). Visualising space and time in crime patterns: A comparison of methods. *Computers, Environment and Urban Systems, 31*(1), 52–75. 10.1016/j.compenvurbsys.2005.07.009.

[CR10] Chan, J. F. W., Yuan, S., Kok, K. H., To, K. K. W., Chu, H., Yang, J., et al. (2020). A familial cluster of pneumonia associated with the 2019 novel coronavirus indicating person-to-person transmission: A study of a family cluster. *The Lancet, 395*(10223), 514–523. 10.1016/S0140-6736(20)30154-9. 10.1016/S0140-6736(20)30154-9 PMC7159286 31986261

[CR12] Cheng, T., Liu, J. X., Zhang, Y., Dong, G., & Liu, Y. (2021). Dynamic spreading of COVID-19 vs community mobility in regions of England. In S. L. Shaw & D. Sui (Eds.), *Mapping COVID-19 in space and time: Understanding the spatial and temporal dynamics of a global pandemic* in press.

[CR13] Demšar, U., & Virrantaus, K. (2010). Space-time density of trajectories: Exploring spatio-temporal patterns in movement data. *International Journal of Geographical Information Science, 24*(10), 1527–1542. 10.1080/13658816.2010.511223.

[CR14] Grantz, K. H., Meredith, H. R., Cummings, D. A. T., Metcalf, C. J. E., Grenfell, B. T., Giles, J. R., Mehta, S., Solomon, S., Labrique, A., Kishore, N., Buckee, C. O., & Wesolowski, A. (2020). The use of mobile phone data to inform analysis of COVID-19 pandemic epidemiology. *Nature Communications, 11*(1), 4961–4961. 10.1038/s41467-020-18190-5. 10.1038/s41467-020-18190-5 PMC7528106 32999287

[CR15] Guan, W., Ni, Z., Hu, Y., Liang, W., Ou, C., He, J., Liu, L., Shan, H., Lei, C. L., Hui, D. S. C., du, B., Li, L. J., Zeng, G., Yuen, K. Y., Chen, R. C., Tang, C. L., Wang, T., Chen, P. Y., Xiang, J., Li, S. Y., Wang, J. L., Liang, Z. J., Peng, Y. X., Wei, L., Liu, Y., Hu, Y. H., Peng, P., Wang, J. M., Liu, J. Y., Chen, Z., Li, G., Zheng, Z. J., Qiu, S. Q., Luo, J., Ye, C. J., Zhu, S. Y., Zhong, N. S., & China Medical Treatment Expert Group for Covid-19. (2020). Clinical characteristics of coronavirus disease 2019 in China. *New England Journal of Medicine, 382*(18), 1708–1720. 10.1056/nejmoa2002032. 32109013 10.1056/NEJMoa2002032 PMC7092819

[CR16] Halloran, M. E., Vespignani, A., Bharti, N., Feldstein, L. R., Alexander, K. A., Ferrari, M., Shaman, J., Drake, J. M., Porco, T., Eisenberg, J. N. S., del Valle, S. Y., Lofgren, E., Scarpino, S. V., Eisenberg, M. C., Gao, D., Hyman, J. M., Eubank, S., & Longini Jr., I. M. (2014). Ebola: Mobility data. *Science*. United States: American Association for the Advancement of Science*, 346*(6208), 433.1. 10.1126/science.346.6208.433-a. 10.1126/science.346.6208.433-a PMC4408607 25342792

[CR17] Hu, S., Xiong, C., Yang, M., Younes, H., Luo, W., & Zhang, L. (2021). A big-data driven approach to analysing and modeling human mobility trend under non-pharmaceutical interventions during COVID-19 pandemic. *Transportation Research Part C: Emerging Technologies, 124*, 102955. 10.1016/j.trc.2020.102955. 33456212 10.1016/j.trc.2020.102955 PMC7796660

[CR19] Jia, J. S., Lu, X., Yuan, Y., Xu, G., Jia, J., & Christakis, N. A. (2020). Population flow drives spatio-temporal distribution of COVID-19 in China. *Nature, 582*(7812), 389–394. 10.1038/s41586-020-2284-y. 32349120 10.1038/s41586-020-2284-y

[CR20] Kang, D., Choi, H., Kim, J. H., & Choi, J. (2020). Spatial epidemic dynamics of the COVID-19 outbreak in China. *International Journal of Infectious Diseases, 94*, 96–102. 10.1016/j.ijid.2020.03.076. 32251789 10.1016/j.ijid.2020.03.076 PMC7194591

[CR21] Kaur, S. P., & Gupta, V. (2020). COVID-19 Vaccine: A comprehensive status report. *Virus Research*. Elsevier BV. 10.1016/j.virusres.2020.198114. 10.1016/j.virusres.2020.198114 PMC7423510 32800805

[CR22] Kupferschmidt, K., & Cohen, J. (2020). Race to find COVID-19 treatments accelerates. *Science*, United States. 10.1126/science.367.6485.1412. 10.1126/science.367.6485.1412 32217705

[CR24] Lu, R., Zhao, X., Li, J., Niu, P., Yang, B., Wu, H., Wang, W., Song, H., Huang, B., Zhu, N., Bi, Y., Ma, X., Zhan, F., Wang, L., Hu, T., Zhou, H., Hu, Z., Zhou, W., Zhao, L., Chen, J., Meng, Y., Wang, J., Lin, Y., Yuan, J., Xie, Z., Ma, J., Liu, W. J., Wang, D., Xu, W., Holmes, E. C., Gao, G. F., Wu, G., Chen, W., Shi, W., & Tan, W. (2020). Genomic characterisation and epidemiology of 2019 novel coronavirus: Implications for virus origins and receptor binding. *The Lancet, 395*(10224), 565–574. 10.1016/S0140-6736(20)30251-8. 10.1016/S0140-6736(20)30251-8 PMC7159086 32007145

[CR25] Nakaya, T., & Yano, K. (2010). Visualising crime clusters in a space-time cube: An exploratory data-analysis approach using space-time kernel density estimation and scan statistics. *Transactions in GIS, 14*(3), 223–239. 10.1111/j.1467-9671.2010.01194.x.

[CR26] Podobnik, B., Wang, D., Horvatic, D., Grosse, I., & Stanley, H. E. (2010). Time-lag cross-correlations in collective phenomena. *EPL, 90*(6), 68001. 10.1209/0295-5075/90/68001.

[CR27] Raboisson, D., & Lhermie, G. (2020). Living with COVID-19: A systemic and multi-criteria approach to enact evidence-based health policy. *Frontiers in Public Health, 8*, 294. 10.3389/fpubh.2020.00294. 32612973 10.3389/fpubh.2020.00294 PMC7308759

[CR28] Santamaria, C., Sermi, F., Spyratos, S., Iacus, S. M., Annunziato, A., Tarchi, D., & Vespe, M. (2020). Measuring the impact of COVID-19 confinement measures on human mobility using mobile positioning data. A European regional analysis. *Safety Science, 132*, 104925. 10.1016/j.ssci.2020.104925. 32952303 10.1016/j.ssci.2020.104925 PMC7486861

[CR29] Siwiak, M., Szczesny, P., & Siwiak, M. (2020). From the index case to global spread: The global mobility based modelling of the COVID-19 pandemic implies higher infection rate and lower detection ratio than current estimates. *PeerJ, 2020*(7), e9548. 10.7717/peerj.9548. 10.7717/peerj.9548 PMC7357567 32728498

[CR30] Vetter, P., Vu, D. L., L’Huillier, A. G., Schibler, M., Kaiser, L., & Jacquerioz, F. (2020). Clinical features of Covid-19. *The BMJ*. England. 10.1136/bmj.m1470. 10.1136/bmj.m1470 32303495

[CR31] World Bank. (2020). *The global economic outlook during the COVID-19 pandemic: A changed world*. World Bank https://www.worldbank.org/en/news/feature/2020/06/08/the-global-economic-outlook-during-the-covid-19-pandemic-a-changed-world.

[CR32] World Health Organization. (2021). *WHO Coronavirus (COVID-19) Dashboard*. WHO https://covid19.who.int/.

[CR33] Xie, J., Song, Z., Li, Y., & Ma, Z. (2018). Mobile big data analysis with machine learning. arXiv preprint arXiv:1808.00803.

[CR34] Xiong, C., Hu, S., Yang, M., Luo, W., & Zhang, L. (2020). Mobile device data reveal the dynamics in a positive relationship between human mobility and COVID-19 infections. *Proceedings of the National Academy of Sciences of the United States of America, 117*(44), 27087–27089. 10.1073/pnas.2010836117. 33060300 10.1073/pnas.2010836117 PMC7959523

[CR35] Yabe, T., Tsubouchi, K., Fujiwara, N., Wada, T., Sekimoto, Y., & Ukkusuri, S. V. (2020). Non-compulsory measures sufficiently reduced human mobility in Tokyo during the COVID-19 epidemic. *Scientific Reports, 10*(1), 18053. 10.1038/s41598-020-75033-5. 33093497 10.1038/s41598-020-75033-5 PMC7581808

[CR36] Zhou, Y., Xu, R., Hu, D., Yue, Y., Li, Q., & Xia, J. (2020). Effects of human mobility restrictions on the spread of COVID-19 in Shenzhen, China: A modelling study using mobile phone data. *The Lancet Digital Health, 2*(8), e417–e424. 10.1016/S2589-7500(20)30165-5. 32835199 10.1016/S2589-7500(20)30165-5 PMC7384783

